# Influence of Sex on Meat and Fat Quality from Heavy Alentejano Pigs Finished Outdoors on Commercial and High Fiber Diets

**DOI:** 10.3390/ani13193099

**Published:** 2023-10-04

**Authors:** José Manuel Martins, Rui Charneca, Nicolás Garrido, André Albuquerque, Eliana Jerónimo, Olinda Guerreiro, Patrícia Lage, Carla Marmelo, Filipa Costa, Amélia Ramos, Luísa Martin

**Affiliations:** 1 MED—Mediterranean Institute for Agriculture, Environment and Development & CHANGE—Global Change and Sustainability Institute, Departamento de Zootecnia, ECT—Escola de Ciências e Tecnologia, Universidade de Évora, Pólo da Mitra, Ap. 94, 7006-554 Évora, Portugal; rmcc@uevora.pt (R.C.); nicolas.osa@uevora.pt (N.G.); andrealb@uevora.pt (A.A.); carla.marmelo@racoessantiago.pt (C.M.); 2 ECO-PIG Consortium, Z.I. Catraia, 3440-131 S. Comba Dão, Portugal; lipaaa.costa@gmail.com (F.C.); ameliaramos@esac.pt (A.R.); luisam@esac.pt (L.M.); 3 Centro de Biotecnologia Agrícola e Agro-Alimentar do Alentejo (CEBAL), Instituto Politécnico de Beja, 7801-908 Beja, Portugal; eliana.jeronimo@cebal.pt (E.J.); olinda.guerreiro@cebal.pt (O.G.); patricia.lage@cebal.pt (P.L.); 4 MED & CHANGE, Centro de Biotecnologia Agrícola e Agro-Alimentar do Alentejo (CEBAL), 7801-908 Beja, Portugal; 5 Departamento de Ciências Agrárias e Tecnologias, Escola Superior Agrária de Coimbra, 3045-601 Coimbra, Portugal

**Keywords:** autochthonous breed, intact pigs, *Longissimus lumborum*, *Psoas major*, dorsal subcutaneous fat, meat and fat quality

## Abstract

**Simple Summary:**

A growing consumer focus on animal welfare, high-quality meat, and product traceability has led to an increased demand for meat from ancient local breeds reared outdoors. Traditionally, these systems involve the slaughter of heavy and fat pigs, which are castrated to mitigate boar taint, an off odor and flavor rejected by consumers when detected. A more welfare-friendly approach involves rearing and slaughtering young intact pigs, leaner and with limited boar taint content in meat and fat. This goal can potentially be achieved through diet manipulation, a strategy that has proven effective in lean industrial breeds but remains unexplored in fatty local breeds like the Portuguese Alentejano pig. In this trial, we studied the influence of gender on meat and fat quality in Alentejano pigs reared outdoors that were fed both commercial and experimental diets during fattening. The results revealed that outdoor finished intact Alentejano pigs produced meat and fat with higher leanness and lower levels of saturation than castrated ones. The LL muscle of intact pigs presented a lower antioxidant activity compared to castrated ones, but this did not affect the lipid oxidative stability of cooked meat between the experimental groups. The experimental diet, with no negative effects on growth, did not influence boar taint compounds, which remained below the threshold values for consumer detection on all Alentejano pigs.

**Abstract:**

This work aimed to examine the effects of sex on meat and fat quality traits from thirty Portuguese Alentejano (AL) pigs reared in outdoor conditions. These pigs were divided into three groups and fed *ad libitum*. From ~40 to 130 kg LW, castrated (C group) and intact animals (I and IExp) consumed commercial diets. Until slaughter (~160 kg), C and I pigs remained on commercial diets, and IExp changed to a more sustainable experimental diet with locally produced pulses and byproducts. Samples were collected from the *Longissimus lumborum* (LL), *Psoas major* (PM), and dorsal subcutaneous fat (DSF). At ~160 kg, the PM muscle of intact pigs presented lower intramuscular fat content than that of C pigs, while total collagen was higher. Additionally, PM myoglobin was lower and lightness (L*) was higher in intact pigs. Regarding DSF, moisture and total protein contents were higher and total lipids were lower in intact than in castrated pigs, while color parameters were not significantly different. Finally, antioxidant capacity measured in the LL muscle showed an overall lower value in intact pigs. However, lipid oxidation values were not significantly different between the experimental groups and only increased with storage time. Outdoor-reared intact AL pigs produced leaner and less saturated pork and fat compared to castrated ones. Despite the lower antioxidant activity observed in the LL muscles of intact pigs, the lipid oxidative stability of cooked meat was not different among the experimental groups.

## 1. Introduction

Consumers’ growing interest in meat and meat products obtained from local breeds [1] can partly be attributed to their rearing and management practices, which allow animals to express natural behaviors, improving animal welfare. Additionally, these products are known for their superior quality and organoleptic characteristics compared to those produced from lean industrial breeds [2]. However, contributing to the maintenance of this quality, these animals are typically slaughtered at an advanced age and high weight. As a result, castration is usually needed to mitigate the potential occurrence of boar taint in the meat and meat products of these animals, which can negatively affect consumer acceptance.

Surgical castration is commonly performed to reduce fat androstenone (AND) and skatole (SKA) levels to below the threshold values, thus preventing boar taint. However, this procedure hinders the growth of pigs, leading to increased production costs [3] and rearing concerns about animal welfare [4]. Moreover, castration encourages fattening and may affect certain characteristics of fresh and cooked meat, such as water retention capacity, color, aroma, taste, juiciness, and tenderness. These characteristics are linked to intramuscular fat content [5,6].

The Alentejano (AL) fatty pig, a local breed from the south of Portugal, belongs to the Iberian type [7] and bears a close genetic resemblance to the Iberian pig [8]. Traditionally reared outdoors, AL pigs are surgically castrated and slaughtered at heavy weights, producing meat and meat products widely acknowledged for their higher quality [4,9]. This breed and its outdoor production system provide economic, ecological, and social added value to their region of origin [9,10]. However, given the current issues involving surgical castration [11,12], alternative solutions with no castration must be explored to prevent or reduce boar taint in animals slaughtered at heavy weights. One solution is diet manipulation, similar to the approach adopted for lean industrial pig breeds.

Intact AL male pigs slaughtered at high live weights exhibited unexpected mean levels of AND and SKA in subcutaneous neck fat [13] below the threshold values employed to distinguish between tainted and untainted carcasses [14]. Nevertheless, apart from the boar taint issue, other meat and fat quality traits may also play a role in meat and meat products’ consumer acceptance and could be altered in intact pigs [15,16]. However, research into these traits, mainly conducted in intact pigs of lean meat-producing industrial hybrids slaughtered at lighter weights, has yielded inconsistent results [17]. Therefore, an open question remains regarding the extent to which using intact local and/or fatter pigs might impact meat and fat quality traits, in addition to their effects on boar taint [18,19].

In this trial, we aimed to compare some quality traits of the *Longissimus lumborum* (LL), *Psoas major* (PM) muscles, and the dorsal subcutaneous fat (DSF). Samples of these tissues were obtained from barrows and intact male AL pigs reared outdoors and fed different diets. The pigs had access to commercial growing–finishing diets, as well as a sustainable high-fiber finishing experimental diet. In conclusion, this study investigates the influence of sex and diet on meat and fat quality traits in heavy intact male AL pigs, contributing to sustainable meat production practices that favor animal welfare and meet consumer demands for high-quality products.

## 2. Materials and Methods

Procedures used in this research were approved by the Bioethical Committee for Animal Experimentation (ORBEA) of Évora University (process GD/38814/2020/P1), Portugal.

The experimental period was characterized by an average temperature of 20.8 °C, average minimal and maximal temperatures of 13.0 and 30.1 °C, and an average relative humidity of 53.4%. All pigs remained in good health throughout this period.

### 2.1. Animals and Experimental Design

The study design was previously detailed in a companion publication [13]. Pure AL male pigs, registered in the herdbook of the breed, were reared in outdoor conditions from ~40 to 160 kg live weight (LW) to investigate the impact of sex and diets on the quality traits of meat and fat. These included the chemical composition, pH, CIE color and fatty acid profiles of *Psoas major* (PM) and dorsal subcutaneous fat (DSF), as well as the antioxidant status and meat oxidative stability of *Longissimus lumborum* (LL). The animals were divided into three experimental groups (*n* = 10 per group) with similar genealogy, body weight, and age. The groups consisted of group C, with pigs that underwent surgical castration under anesthesia and analgesia, and groups I and IExp, comprising non-castrated (intact) boars. Each group was reared in a 1000 m^2^ park, featuring stalls with individual feeders and inox drinking nipples, a zinc shelter (16 m^2^) and scattered trees. During summer, a water pond (~6 m^2^) was made available in each park to aid in thermoregulation and improve welfare. Between ~40 and 130 kg LW, pigs had access to commercially sold breed-specific growing and fattening diets at an estimated *ad libitum* intake [9]. Between ~130 and 160 kg, groups C and I continued to consume the commercial fattening diet. However, the IExp group received an isoenergetic and isoproteic experimental diet (digestible energy ≈ 13.1 MJ/kg DM, CP ≈ 14%) containing beet, pulses such as lupin and peas, and locally produced malt rootlets (see Supplementary Table S1 [20,21,22,23,24,25]). The selection of ingredients for the experimental diet was based on documented evidence of their ability to reduce boar taint compounds in the fat of intact industrial pigs, as cited in the literature. The specific formulations of the commercial and the experimental diets are proprietary information. Diets were provided once daily (at 9:00 h) until the onset of summer, when they were fed twice daily (at 9:00 and 19:00 h). Daily feed consumption was adjusted weekly and water was provided ad libitum. Diet refusals were registered on a daily basis.

After ~27 weeks of trial, the AL pigs were slaughtered at an average of 157.1 ± 1.3 kg LW at an industrial slaughterhouse. Animals were stunned with carbon dioxide just before bleeding. Each experimental group was housed in a separate pen, and pigs were killed after a ~12 h fast during lairage, but with free access to water. After slaughter, samples from LL, PM, an important cut in the Portuguese fresh meat market, and DSF (outer and inner layers) were collected from the left-side carcasses. These samples were vacuum-packaged and subsequently frozen (−80 and −20 °C) until analyses.

### 2.2. Diets Analyses

The analyses of diets were previously described in a companion paper [13]. Briefly, commercial and experimental diets were analyzed for dry matter (UE 500, Memmert, Schwabach, Germany), total ashes, crude protein (N × 6.25) (Kjeldatherm KB-20, Gerhardt, Bonn, Germany, and Kjeltec Auto 1030 Analyzer, Tecator, Bristol, UK), neutral and acid detergent fibers, total and insoluble fibers, and total sugars [22]. Cellulose and total starch were determined according to ISO-6865 [23] and ISO-6493 [24], respectively. The total lipids were quantified with a Soxtherm automatic apparatus (SE416; Gerhardt, Bonn, Germany), and the fatty acids (FA) were analyzed from lipid extracts, in accordance with ISO-12966-4 [26].

### 2.3. Muscle and Fat Analyses

The moisture content of PM and DSF samples was determined following the ISO-1442 [27]. Total nitrogen in PM (method 992.15) and DSF (method 928.08) was analyzed using the Dumas combustion method [22] with a Leco FP-528 Nitrogen/Protein Determinator (Leco Corp., St. Joseph, MI, USA) and the crude protein content was calculated (N × 6.25). The intramuscular fat (IMF) content in PM was analyzed according to Folch et al. [28], while total lipids in DSF were quantified through Soxhlet extraction (method 991.36 [22]). The fatty acids (FAs) extracted from PM and DSF [28] were transesterified into methyl esters [29]. For their identification and profiling, a Shimadzu GC-MS2010 Plus chromatograph (Kyoto, Japan), equipped with a capillary column SP-2560 (100 m × 0.25 mm I.D., 0.20 μm) (Supelco, Bellefonte, PA, USA) was used. Lastly, the iodine values of PM and DSF were calculated according to Lo Fiego et al. [30], while the peroxidizability index was calculated according to Witting [31].

PM total ashes were determined in a muffle furnace by carbonization and incineration (550 °C). The ultimate pH (pHu) was measured following ISO-2917 [32] with a pH meter and a puncture electrode (LoT406-M6-DXK-S7/25, Mettler-Toledo GmbH, Gießen, Germany). Haem pigment concentration was determined according to Hornsey [33] and multiplied by a factor of 0.026 [34] to obtain myoglobin content. Total collagen was calculated by multiplying total hydroxyproline [35] by a factor of 7.14 [36]. As for soluble collagen, it was determined according to Hill [37].

The objective color [38] of raw PM (after 30 min of blooming) and DSF samples was measured using a CR-400 colorimeter (Konica Minolta Sensing Europe B.V., Nieuwegein, The Netherlands) equipped with a D-65 illuminant. On average, six random readings across each sample surface were used to obtain the L* (lightness), a* (redness), and b* (yellowness) values. Chroma, hue angle, and saturation were calculated using the following equations: chroma (C) = √(a*^2^ + b*^2^); hue angle (H°) = tan^−1^(b*/a*); saturation = C/L*.

### 2.4. Muscle Antioxidant Status and Meat Oxidative Stability

The overall antioxidant status of muscle from AL pigs was measured using Folin–Ciocalteu, Trolox equivalent antioxidant capacity (TEAC), and ferric reducing antioxidant power (FRAP) assays. An extract was previously prepared where 500 mg of LL was added to 10 mL of distilled water and homogenized for 60 s at 9500 rpm with an Ultra-Turrax T25 homogenizer (IKA Werke GmbH & Co. KG, Staufen, Germany) and then for 6 min (twice for 3 min, with a break of 2 min) in an ultrasound bath (Bransonic^®^ Ultrasonic 3510E-DTH, Branson Ultrasonics Corporation, Danbury, CT, USA). The homogenates were centrifuged at 3000× *g* for 15 min at 4 °C. The supernatant was filtered through Whatman 541 filter paper and stored in microtubes at −80 °C for analysis of overall antioxidant status. The FRAP, TEAC and Folin–Ciocalteu assays were performed following the procedures described by Luciano et al. [39].

Lipid oxidation over storage time was evaluated in cooked LL samples stored for 0, 2, and 4 days after cooking. Three subsamples of vacuum-packed meat were cooked for 30 min in a water bath at 70 °C. Immediately after cooking, lipid oxidation was measured in one sub-sample, while the other subsamples were placed in a polystyrene tray, wrapped with oxygen-permeable polyvinyl chloride film, and stored at 4 °C for 2 or 4 days in the dark. At each sampling time (0, 2, and 4 days), the meat lipid oxidation was determined through the quantification of thiobarbituric acid reactive substances (TBARS) according to the method of Grau Guardiola, Boatella, Barroeta, and Cordony (2000), as described in Francisco et al. [40]. The absorbance of the samples was measured by a double-beam UV–Vis scanning spectrophotometer (Helios Alpha spectrophotometer, Thermo Scientific, Bremen, Germany) at 532 nm. The assay was calibrated using a standard curve, with solutions of 1,1,3,3-tetraethoxypropane of known concentrations. The results were expressed as mg of malonaldehyde (MDA)/kg of meat.

### 2.5. Calculations and Data Analyses

The Shapiro–Wilk test for normality was used to test all data. Results are presented as means ± standard errors (SE). Statistical analysis of data from meat and fat composition and quality was performed by one-way analysis of variance (ANOVA) and that of data for MDA on days 0, 2 and 4 after cooking was performed using a repeated measurements model. The model considered the effects of treatment, day, and interaction of day with treatment, and the autoregressive (AR(1)) covariance structure was used. The treatment × day interaction was not significant. Several Pearson correlation coefficients were calculated between variables. The statistical software used was IBM SPSS Statistics (IBM SPSS Statistics for Windows, v24.0, IBM Corp., Armonk, NY, USA). When *p* < 0.05, differences were considered significant, and *p*-values between 0.05 and 0.10 were considered trends.

## 3. Results

### 3.1. Tissue Physical—Chemical Composition

The PM muscle’s physical—chemical composition was significantly affected by experimental treatments (Table 1). Moisture content was higher in intact (I and IExp) compared to C pigs and showed an inverse relationship with total IMF content. Both intact groups presented lower IMF levels compared to C (−19.2 in I and −25.4% in IExp) (*p* < 0.0001). Total protein and ash contents were not influenced by treatments. Total collagen was also higher in intact pigs (+16.4% in I and IExp) (*p* < 0.001). However, while following the same trend, soluble collagen content in intact pigs was not significantly different from that observed in C pigs. Additionally, myoglobin and color parameters were affected by the experimental treatments. Both intact groups presented lower myoglobin content (*p* = 0.003) (−13.8 in I and −16.6% in IExp), and higher lightness (L*) values (*p* < 0.001) (+5.0 in I and +5.8% in IExp) than in C pigs. Chroma and hue angle were not affected by experimental treatments, while saturation tended to be lower (*p* = 0.096) in intact (−8.8% in both groups) than in C pigs (Table 1).

Dorsal subcutaneous fat moisture, total protein, and total lipids were affected by treatments (Table 2). As observed in PM, DSF moisture showed an inverse relationship with total lipids content and was higher (*p* < 0.0001) in intact (+88.9 in I and +84.4% in IExp) than in C pigs. DSF from both intact groups exhibited higher total protein levels (*p* < 0.0001) (~3 in I and ~2.9 times in IExp) while total lipids were lower (*p* = 0.004) (−14.2 in I and −11.5% in IExp) compared to C pigs. Regarding color parameters, lightness, redness (a*), yellowness (b*), chroma, hue angle, and saturation remained unaffected by treatments (Table 2).

### 3.2. Muscle Fatty Acids Profile

Out of the 26 FAs measured in the IMF of AL pigs, only 7 exceeded the proportion of 1 g/100 g. Across all the animals studied, the most abundant FA found in the intramuscular lipids of PM was oleic acid (C18:1 *n*-9), followed by palmitic (C16), linoleic (C18:2 *n*-6) and stearic acid (C18). Together, these four FAs accounted for over 87% of the total FAs. The content of oleic acid varied from 38.2 to 41.4 g/100 g of total FA methyl esters (FAMEs) analyzed, with the highest proportion observed in IExp and the lowest in C pigs (Table 3).

The experimental treatments significantly affected the proportion of the main FAs detected in the IMF of PM muscle (Table 3). The saturated fatty acid (SFA) proportion was lower (*p* = 0.021) in I (−3.9%) compared to C pigs, with IExp showing intermediate values. This difference was mainly due to a lower proportion of myristic (C14) (*p* < 0.0001) and palmitic acid (*p* < 0.001). Both intact groups presented a lower proportion (*p* < 0.001) of monounsaturated fatty acids (MUFA) (−4.8 in I and −8.1% in IExp) than C pigs. This was primarily attributed to reduced proportions of palmitoleic (C16:1 *n*-7) and cis-vaccenic (C18:1 *n*-7) (*p* < 0.0001) acids, and partly to a 7.7% oleic acid proportion reduction in the IExp group (*p* = 0.001). Finally, the proportion of polyunsaturated fatty acids (PUFA) was higher (*p* < 0.0001) in intact (+23.9 in I and +30.3% in IExp) than in C pigs, largely due to higher proportions of linoleic (*p* < 0.0001) and alpha-linolenic acids (C18:3 *n*-3) (*p* < 0.001) (Table 3). This led to a higher PUFA to SFA ratio (*p* < 0.0001) in intact pigs (+27.9 in I and +32.6% in IExp) when compared to C ones. The *n*-6 FA proportion and the *n*-6 to *n*-3 ratio were also higher (*p* < 0.0001) in both intact (+19.5 in I and +25.7% in IExp) compared to C pigs. Additionally, the saturation and atherogenic indexes were lower (*p* = 0.020 and *p* < 0.001, respectively) in both intact (−5.4 and −3.6% in I, and −8.9 and −6.7% in IExp, respectively), while the iodine value was higher (*p* < 0.0001) (+6.8 and +5.1% in I and IExp, respectively) than in C pigs. Finally, the peroxidizability index was 16.7% higher in I and 13.4% higher in IExp pigs compared to C ones (see Table 3).

### 3.3. Dorsal Subcutaneous Fat Fatty Acids Profile

Out of the 28 FAs measured in DSF, 8 exceeded the proportion of 1 g/100 g. Across all the animals studied, the most abundant FA found in the DSF was oleic acid, followed by palmitic, stearic, and linoleic acid, representing over 91% of the total FAs. Oleic acid was the most prevalent FA detected in the DSF of AL pigs, ranging from 47.1 to 48.4 g/100 g (C and I pigs, respectively) of total FAMEs analyzed (Table 4).

The main proportions of FA in the DSF of AL pigs were affected by the experimental treatments. Similar to the PM muscle, the proportion of SFA was lower (*p* < 0.0001) in both intact groups (−8.2 in I and −9% in IExp) compared to C pigs. This could be attributed to lower proportions (*p* < 0.0001) of myristic, palmitic, and stearic acids. In contrast to the findings in PM, the proportion of MUFA was higher (*p* = 0.008) in both intact groups (+3.3 in I and +3.5% in IExp) than in C pigs, mostly as a result of the higher proportions of hexadecenoic (C16:1 *n*-9, *p* < 0.0001) and oleic acid (*p* = 0.002). Furthermore, as was observed in the muscle, the proportion of PUFA was also higher (*p* < 0.0001) in both intact groups (+13.2 in I and +17.6% in IExp) than in C pigs, mainly as a result of the higher proportion (*p* < 0.0001) of linoleic acid (see Table 4). This led to a higher PUFA to SFA ratio (*p* < 0.0001) in intact pigs (+21.7 in I and +26.1% in IExp) compared to C ones. The proportion of *n*-6 FA and the *n*-6 to *n*-3 ratio were also higher (*p* < 0.0001 and *p* = 0.004, respectively) in intact (+21.7 in I and +24.6% in IExp, and +20 in I and +31.4% in IExp, respectively) than in C pigs. Saturation and atherogenic indexes presented lower values (*p* < 0.0001) in both intact groups (−12.1 in I and −13.6% in IExp, and −11.3 in I and −9.9% in IExp, respectively), while the iodine value was higher (*p* < 0.0001) in both intact groups (+6.1 in I and +7.3% in IExp, respectively) than in C pigs. Finally, the peroxidizability index was 11.4% higher in I and 10.5% higher in IExp pigs compared to C ones (see Table 4).

### 3.4. Loin Antioxidant Status and Meat Oxidative Stability

Compared to the samples from C pigs, LL samples from intact AL pigs exhibited lower levels of total phenols (−16.1 for I and −19.6% for IExp), and Trolox equivalent antioxidant capacity (TEAC) (−19 for I and −16.7% for IExp) (Table 5). However, in contrast to the findings of total phenols and TEAC, the ferric-reducing antioxidant power (FRAP) was not affected by the experimental treatments.

**Figure 1:**
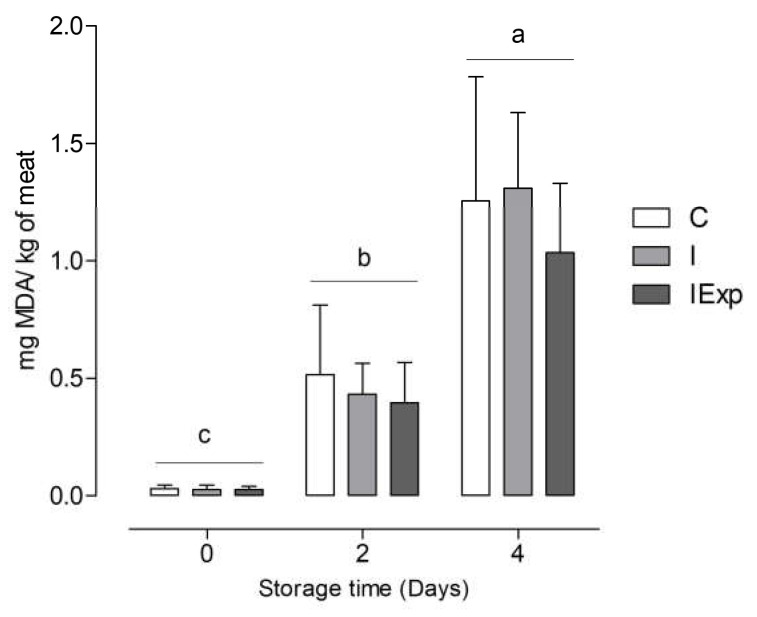
Lipid oxidation over storage time in cooked LL samples from castrated (C), intact (I) and intact experimental (IExp) Alentejano pigs slaughtered at ~160 kg LW (*n* = 10 per group; ^a,b,c^ Columns with different letters are significantly different (*p* < 0.05)).

Lipid oxidation, evaluated in cooked LL samples stored for 0, 2, and 4 days after cooking, showed a significant increase with longer storage duration. However, within each storage period, there were no significant differences observed in lipid oxidation between the samples from C, I, and IExp pigs (Figure 1).

## 4. Discussion

Current knowledge suggests that sex influences more than just boar taint compounds. It also affects meat and fat quality traits, which influence consumer acceptance of meat and meat products derived from intact animals [15,16]. Research into these effects is usually conducted using industrial hybrid pigs selected for their lean meat characteristics, but findings are not always consistent [17]. Intact males may offer a more sustainable option concerning animal welfare, socio-economical, and environmental aspects, as long as AND and SKA levels are not detected by consumers [19]. So, it is vital to study the impact of sex on meat and meat product quality alongside consumer acceptance. This trial is the first to use fatty AL pigs reared outdoors with *ad libitum* access to commercial and experimental diets. Its objective was to investigate meat and fat quality traits in castrated and intact animals, killed at ~160 kg body weight. Additionally, despite its high consumer demand [41], the PM muscle remains understudied.

**Figure 2:**
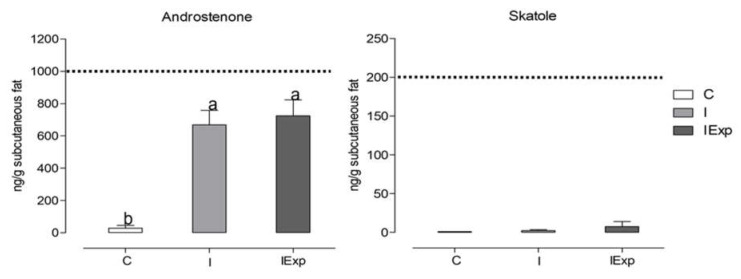
Androstenone and skatole content of neck subcutaneous fat from castrated (C), intact (I) and intact experimental (IExp) Alentejano pigs slaughtered at ~160 kg LW [13]. (*n* = 10 per group; 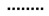 Threshold values for consumer detection [14]; ^a,b^ Columns with different letters are significantly different (*p* < 0.05)).

Growth data, carcass characteristics, LL physical–chemical traits, and fat AND and SKA contents of the AL pigs used in this trial were previously discussed in a companion paper by Martins et al. [13]. Briefly, intact pigs (I and IExp) showed faster growth, improved feed conversion ratios, lower carcass yields, and leaner carcasses with higher commercial yields and lower proportions of fat cuts compared to castrated pigs. The LL muscle of intact pigs had lower intramuscular fat content, which was less saturated and more polyunsaturated, along with higher total collagen content. AND and SKA fat content were also presented in the companion paper [13]. While intact pigs had higher fat AND content than C ones, the SKA content was similar across all treatments (Figure 2). Feeding the experimental diet to IExp pigs had no effect on fat SKA content compared to pigs fed the commercial diet, as discussed in [13]. However, it should be noted that both AND and SKA levels remained below the threshold values for consumer detection in all experimental groups.

To the best of our knowledge, apart from the companion study by Martins et al. [13], no bibliographical information is available regarding intact AL or Iberian male pigs slaughtered at heavy weights. In this trial, AL pigs were killed at an average age of 294.4 days, with a mean body weight of 157.1 kg. Moreover, previous studies examining the effects of sex in industrial pigs typically focused on parameters such as growth, feed efficiency, and carcass composition [42].

In this trial, the chemical and physical composition of the muscle was affected by sex. The moisture and protein content of PM in C pigs were similar to those found in *ad libitum*-fed castrated Iberian pigs slaughtered at 160–180 kg LW [43]. Still, the IMF content was lower, probably due to differences in age/weight at slaughter. Both intact groups, besides presenting reduced body fat and LL IMF content [13], also showed lower IMF content in the PM muscle compared to C pigs. The pH values of the PM in all experimental groups remained within the typical meat pH range, which varies from 5.5 to 5.8 [44], and were not influenced by the experimental treatments.

Meat color is widely recognized as a crucial visual factor influencing meat quality [45]. In contrast to LL muscle results [13], sex affected the color attributes of PM muscle. Specifically, L* values were significantly higher in intact groups (I and IExp) than in C pigs. This indicates a lighter meat color in intact pigs, which agrees with the lower myoglobin content in the PM muscle of intact when compared to C pigs. This suggests differences in the visual perception of PM color in intact versus castrated AL pigs, unlike the findings in the LL muscle [13]. However, although redness (a*) was higher in C than in intact groups, it did not attain statistical significance. Finally, the myoglobin content in the more oxidative PM muscle was higher than in the glycolytic LL muscle [13], as reported in crossbred industrial pigs [46].

Intact males often have slightly higher collagen content in muscle tissues compared to castrated pigs. This could be due to increased collagen synthesis and more soluble collagen could potentially be present due to the anabolic effect of testosterone [6]. In the LL [13] and PM muscles of intact AL pigs, total collagen content was higher than in C pigs, but an increase in soluble collagen was not observed, as reported in industrial pigs [15].

Regarding the physical–chemical composition of DSF in AL pigs, only chemical traits were affected by sex. Moisture, total protein and total lipid content of DSF in C pigs were similar to those found in castrated AL pigs also killed at ~160 kg LW [47]. Still, moisture was higher, while lipid content was lower in intact (I and IExp) than in C pigs, confirming the negative correlation between these parameters [48]. Total protein content in DSF was higher in intact pigs, as previously observed in industrial pigs [48,49], likely due to increased fat deposition in C pigs, primarily through adipocyte hypertrophy [50]. Although histomorphology and cellularity studies on intact pigs’ adipose tissue are limited [11], it has been reported that surgically castrated pigs have larger and fewer adipose cells than intact ones [51]. Thus, the lower protein concentration in C pigs may be explained by reduced adipose tissue cellularity.

Sex influenced muscle FA composition. Intact pigs presented lower SFA and MUFA, and higher PUFA levels than C ones, a pattern consistent with LL muscle results [13] and a meta-analysis [52] in industrial breeds. Reduced SFA and MUFA levels in leaner pigs could be due to decreased *de novo* synthesis and FA turnover [53,54], potentially linked to post-puberty increases in anabolic steroids [55].

As in industrial pigs [11,54], the lower SFA proportion observed in the PM of intact AL pigs was mainly due to a reduction in palmitic acid. Notably, this decline in SFA was significant only between the C and I groups. A higher PUFA content in the commercial diet than in the experimental one (1.77 vs. 1.66 g/100 g DM—Table S1 [20,21,22,23,24,25]) might have influenced this difference through a reduced *de novo* FA synthesis [56]. Additionally, a greater feed intake in I compared to IExp pigs may have further suppressed *de novo* FA synthesis in the former group. The reduction in SFA (and increase in PUFA) also impacted the saturation index in intact groups, with positive implications for consumer health [57]. The lower proportion of MUFA in intact pigs compared to castrated ones resulted from a reduction in oleic (significantly different between C and IExp pigs), cis-vaccenic (C18:1 *n*-7) and palmitoleic acid (C16:1 *n*-7). Oleic acid content is influenced by the *de novo* elongation and desaturation of stearic acid, primarily mediated by stearoyl-CoA desaturase (SCD) activity [58]. This enzyme activity was not directly measured at this trial, but desaturation indexes (palmitoleic to palmitic acid and oleic to stearic acid) serve as indicators of SCD activity [59]. In the IMF of PM, intact groups showed lower desaturation indexes for palmitoleic to palmitic acid (*p* = 0.003) and oleic to stearic acid (*p* = 0.083). This suggests reduced SCD activity in these groups, as reported in previous studies comparing barrows and non-castrated males [16,54,60]. Moreover, intact pigs are known for their higher physical activity and social interactions [61]. Increased oleic acid oxidation in the muscles of these pigs, a readily available energy source [62], may have contributed to reducing MUFA levels. The PUFA content, particularly linoleic acid, was higher in the IMF of both intact groups than in C pigs, as reported in industrial pigs [11,52], which is relevant for consumer health. The observed lower PUFA content in C pigs may be due to a dilution effect [63]. In fact, castrated pigs have greater *de novo* lipogenic activity than intact ones [64], reducing PUFA proportions in C pigs, which are obtained from the diet [63]. The differences in PUFA between experimental groups led to an increased content of *n*-3 (*p* = 0.103) and *n*-6 FAs (*p* < 0.0001) in both intact groups. These *n*-3 and *n*-6 PUFA shifts are known to redirect FAs from triglyceride synthesis toward oxidation (reviewed by [65]), which agrees with the lower blood triacylglycerol content and body fat deposition observed in intact when compared to C pigs [13]. Additionally, the increase in linoleic acid and the decrease in docosahexaenoic acid (C22:6 *n*-3) led to a significant rise in the *n*-6 to *n*-3 ratio of intact pigs compared to C ones. Recognized as a risk factor for coronary heart diseases [63], the values detected in the IMF of PM from these AL pigs are favorably compared to the ones from industrial pigs [66]. Lastly, both IMF content and FA profile of the more oxidative PM muscle differed from those of the glycolytic LL muscle [13]. The PM presented lower IMF content and MUFA levels, and higher PUFA proportions than the LL muscle, as reported in industrial pigs [46,67].

In this trial, DSF FAs were also influenced by sex. Overall, intact pigs presented lower SFA, mainly due to decreased palmitic and stearic acids, and higher PUFA proportions than castrated pigs, as previously reported in industrial breeds [54,60]. As in the PM muscle, the lower SFA proportion in leaner intact pigs may be due to reduced *de novo* synthesis and FA turnover [54] linked to post-puberty testosterone increases [55]. Regarding PUFA, its lower proportion in C pigs could also be attributed to a dilution effect, as in the PM muscle. However, while both tissues saw an increase in linoleic acid, DSF presented about half the PUFA difference seen in the muscle. This variation suggests a more efficient inhibition of SCD activity in PM muscle than in DSF of intact pigs, partly explaining oleic acid deposition differences. In line with the concept of independent FA synthesis regulation in muscle and fat [68], intact pigs presented higher MUFA proportions in DSF, mainly due to an increase in oleic acid, which contrasts with muscle findings. This increased MUFA content aligns with reduced DSF thickness observed in intact pigs [13]. The inverse correlation between DSF thickness and lipid unsaturation observed (r = −0.561, *p* = 0.004) aligns with earlier reports [69]. Also, testosterone administration in rats was linked to increased SCD expression and activity using both palmitoyl-CoA and stearoyl-CoA as substrates [70]. This could have contributed to the lower content of palmitic and stearic acids observed in intact pigs (see above). Nonetheless, mixed results exist in industrial breeds regarding MUFA contents in DSF, with some studies reporting higher content in castrated pigs [11,49], and others finding no significant differences [14,69]. These discrepancies may be linked to differences in diets, sampling, analytical techniques, and other factors. For instance, the inner DSF layer is typically more saturated due to preferential PUFA deposition in the outer layer and stimulated *de novo* lipogenesis in the inner layer [71]. Genetic background may also play a role, partly explaining the differences between AL and industrial breeds, as AL is known to synthesize higher oleic acid proportions in muscle and subcutaneous fat [66,72]. Additional studies are required to elucidate this matter further.

The FA composition influences pork and fat quality, namely fat firmness (due to the different melting points of meat FAs), shelf life (due to their propensity to oxidize) and flavor [67]. As observed in LL [13], besides the lower IMF content, PM muscles from intact males also presented a distinct FA composition. These differences in pork from intact and C pigs led to a healthier PUFA to SFA profile, a reduction in saturation and atherogenic indexes, potentially lowering coronary heart disease risk [57]. However, the higher linoleic PUFA content of pork raises concerns due to its increased oxidation susceptibility [5,73] and potential for off flavors [74]. Additionally, iodine values, indicating unsaturation degree in fats and oils and used to estimate oxidative stability and firmness [30], were higher in intact pigs’ PM and DSF (see Table 3 and Table 4). These traits could potentially affect processing and product shelf life, which are of particularly important significance in a breed renowned for its PDO and PGI meat and meat products.

The primary non-microbial cause of meat and meat product quality deterioration is oxidation, an undesirable process that is particularly worrying in foods with high amounts of unsaturated fat [75] such as AL pig meat. During shelf life and storage, oxidative processes can lead to the degradation of pigments, lipids, and proteins. This adversely affects color, texture, and nutritional value, while also causing the development of off flavors and odors [51,76,77]. Factors such as muscle type, anatomical location, species, breed, rearing system, and diet can influence oxidation and significantly alter meat attributes, composition, and nutritional value [75,78]. FAs are the main substrate for oxidation in meat. The level of unsaturation in FAs, namely high levels of PUFA, has a negative impact on oxidative stability and shelf life [51,73]. Accordingly, the higher levels of PUFA observed in the LL of intact pigs compared to castrated pigs, as reported in a companion paper [13], may increase meat’s susceptibility to lipid oxidation in the former pigs. Similarly, the LL peroxidability index, based on its FA composition [13] and calculated according to Witting [31], was higher in intact pigs (10.3, 14.6 and 13.7 in C, I and IExp pigs, respectively, *p* < 0.001). This trend was also observed in PM and DSF tissues. However, oxidation reactions depend on the balance between pro-oxidant and antioxidant factors, which ultimately determine the oxidative stability of meat. Beet, pulses and malt rootlets are recognized for their richness in bioactive compounds with antioxidant activity [79,80], suggesting that including them in the experimental diet may enhance meat antioxidant activity and oxidative stability in IExp pigs.

In addition to assessing oxidation levels through conventional or advanced techniques [77], there is a growing interest in evaluating meat and meat products’ antioxidant status. Antioxidant capacity tests are particularly useful for assessing the antioxidant status of meat from animals fed different diets, although their implementation is still limited [78]. While there is no single method that comprehensively addresses all aspects of antioxidant capacity in meat and meat products, there are multiple evaluation methods available, providing complementary information. In our trial, LL antioxidant status, evaluated by Folin–Ciocalteu and radical scavenging ability (TEAC assay), was significantly lower in both intact groups than in C pigs. However, the LL-reducing ability (FRAP assay) was not affected by experimental treatments. These different antioxidant evaluation methods involve various antioxidant mechanisms [78], often giving inconsistent results between assays, as observed in the literature. Overall, the variations in total phenols and TEAC levels suggest variations in the global antioxidant capacity between intact and C pigs. On the other hand, nonsignificant FRAP differences may indicate that the specific antioxidants involved in the reduction in ferric ions are similar between the three groups. Nevertheless, the dietary inclusion of pulses and agro-industrial byproducts did not improve the antioxidant status in LL from IExp pigs. Only a reduction in antioxidant activity was observed in LL from pigs of both intact groups. This reduction may be related to the greater utilization of antioxidant compounds to respond to oxidative pressure induced by the higher levels of FAs highly susceptible to oxidation. Despite the higher susceptibility of lipid oxidation and lower antioxidant activity in LL muscles from pigs of both intact groups, the lipid oxidative stability of cooked meat was not affected by sex or diet (see Figure 1). This indicates that pork from all treatments can respond equally to increased oxidative pressure induced by cooking, leading to similar TBARS value increases during storage. Furthermore, LL samples from all experimental groups did not exceed the 2 mg MDA/kg of meat, considered the threshold value at which rancidity may be detected by consumers in meat and meat products [51]. Regarding the LL, PM and DSF iodine values of pigs from all experimental groups, only the PM muscle of I pigs showed averages ≥ 70. This value is used in Danish works as an indicator of soft fat with poor keeping quality [49]. Fat firmness is crucial in fresh meat marketing, influencing appearance and ease of handling [81]. Among the FAs, stearic acid has the strongest correlation with pig fat firmness, followed by linoleic acid. However, from the perspective of meat marketing, the latter holds greater significance [82].

## 5. Conclusions

In this work, intact pigs from the AL breed reared outdoors and consuming a sustainable finishing diet based on locally produced pulses and agro-industrial byproducts, were compared to intact and castrated AL fed a commercially available diet. As previously reported [13], boar taint compounds detected in the subcutaneous fat of AL pigs were below the threshold values for consumer detection. Overall, our data showed that intact pigs produced leaner, lighter, and more (poly)unsaturated meat than castrated ones. The same trends were observed on subcutaneous fat tissues where, interestingly, oleic acid content was higher in intact than in C pigs. Despite the higher susceptibility of lipid oxidation and lower antioxidant activity observed in the LL of intact pigs, these differences did not lead to variations in the lipid oxidative stability of cooked meat among the experimental groups. Additionally, based on I and IExp pig’s data, the experimental diet did not present major adverse effects on meat and fat quality traits. Further research with larger sample sizes is needed to confirm these findings and assess the implications of using meat and fat from intact AL fatty pigs in products like PDO qdry-cured hams, which hold significant economic importance in this breed’s production system.

## Figures and Tables

**Table 1:** Chemical composition, pH, and CIE color values of *Psoas major* from castrated (C), intact (I) and intact experimental (IExp) Alentejano pigs slaughtered at ~160 kg LW (*n* = 10 per group) ^#^.

	C		I		IExp		*p*-Value
	Mean	SE	Mean	SE	Mean	SE	
Moisture (g/100g)	73.5 ^b^	0.2	74.4 ^a^	0.2	74.5 ^a^	0.2	<0.001
Total protein (g/100g)	22.6	0.1	22.3	0.2	22.3	0.1	0.175
Total intramuscular fat (g/100g)	2.40 ^a^	0.10	1.94 ^b^	0.05	1.79 ^b^	0.09	<0.0001
Total ashes (g/100g)	1.18	0.01	1.19	0.01	1.18	0.02	0.798
pH (24 h *post mortem*)	5.66	0.02	5.67	0.02	5.65	0.02	0.893
Total collagen (mg/g DM)	15.2 ^b^	0.3	17.7 ^a^	0.4	17.7 ^a^	0.5	<0.001
Soluble collagen (mg/g DM)	4.05	0.19	4.29	0.15	4.18	0.13	0.548
Myoglobin content (mg/g)	3.19 ^a^	0.09	2.75 ^b^	0.12	2.66 ^b^	0.11	0.003
Lightness (L*)	36.4 ^b^	0.3	38.2 ^a^	0.4	38.5 ^a^	0.4	0.001
Redness (a*)	18.5	0.5	17.9	0.7	17.8	0.6	0.665
Yellowness (b*)	8.9	0.4	8.7	0.3	8.8	0.3	0.846
Chroma (C)	20.6	0.7	19.9	0.6	19.9	0.6	0.661
Hue angle (H°)	25.7	0.7	26.0	1.0	26.4	1.0	0.872
Saturation	0.57	0.02	0.52	0.02	0.52	0.02	0.096

^#^ C and I pigs were fed commercial diets until slaughter, while IExp pigs consumed commercial diets until ~130 kg and then the experimental diet until slaughter; ^a,b^ Values with different superscript letters in the same row are significantly different (*p* < 0.05).

**Table 2:** Chemical composition, and CIE color values of dorsal subcutaneous fat from castrated (C), intact (I) and intact experimental (IExp) Alentejano pigs slaughtered at ~160 kg LW (*n* = 10 per group) ^#^.

	C		I		IExp		*p*-Value
	Mean	SE	Mean	SE	Mean	SE	
Moisture (g/100g)	4.5 ^b^	0.3	8.5 ^a^	0.6	8.3 ^a^	0.5	<0.0001
Total protein (g/100g)	0.68 ^b^	0.12	2.06 ^a^	0.20	1.94 ^a^	0.19	<0.0001
Total lipids (g/100g)	94.1 ^a^	0.8	80.7 ^b^	3.4	83.3 ^b^	3.1	0.004
Lightness (L*)	82.8	0.3	83.4	0.4	83.3	0.1	0.367
Redness (a*)	2.22	0.14	2.24	0.20	2.37	0.14	0.771
Yellowness (b*)	4.53	0.16	4.77	0.14	4.84	0.13	0.301
Chroma (C)	5.05	0.20	5.29	0.19	5.39	0.18	0.425
Hue angle (H°)	64.0	1.0	65.2	1.7	64.1	0.8	0.732
Saturation	0.06	0.01	0.06	0.01	0.07	0.01	0.509

^#^ C and I pigs were fed commercial diets until slaughter, while IExp pigs consumed commercial diets until ~130 kg and then the experimental diet until slaughter; ^a,b^ Values with different superscript letters in the same row are significantly different (*p* < 0.05).

**Table 3:** Main fatty acids profile of intramuscular lipids of *Psoas major* from castrated (C), intact (I) and intact experimental (IExp) Alentejano pigs slaughtered at ~160 kg LW (*n* = 10 per group) ^#^.

	C		I		IExp		*p*-Value
	Mean	SE	Mean	SE	Mean	SE	
	g/100 g of total fatty acids identified						
C14	1.03 ^a^	0.02	0.88 ^b^	0.02	0.91 ^b^	0.03	<0.0001
C16	24.1 ^a^	0.2	22.7 ^b^	0.2	22.9 ^b^	0.3	0.001
C18	10.6	0.2	10.7	0.2	11.1	0.19	0.134
C20	0.111	0.004	0.110	0.006	0.113	0.005	0.904
Σ SFA	36.3 ^a^	0.4	34.9 ^b^	0.2	35.6 ^a b^	0.4	0.021
C16:1 *n*-7	2.57 ^a^	0.03	2.02 ^b^	0.10	2.05 ^b^	0.09	<0.0001
C16:1 *n*-9	0.44 ^b^	0.02	0.53 ^a^	0.03	0.51 ^a b^	0.03	0.048
C18:1 *n*-7	3.61 ^a^	0.04	3.13 ^b^	0.07	3.23 ^b^	0.05	<0.0001
C18:1 *n*-9	41.4 ^a^	0.5	40.0 ^a^	0.3	38.2 ^b^	0.7	0.001
Σ MUFA	48.2 ^a^	0.5	45.9 ^b^	0.4	44.3 ^b^	0.8	<0.001
C18:2 *n*-6	11.4 ^b^	0.4	14.9 ^a^	0.3	15.7 ^a^	0.6	<0.0001
C18:3 *n*-3	0.91 ^b^	0.02	1.01 ^a^	0.02	0.99 ^a^	0.01	<0.001
C20:2 *n*-6	0.23 ^b^	0.01	0.30 ^a^	0.01	0.29 ^a^	0.01	<0.0001
C20:4 *n*-6	2.06	0.12	2.07	0.09	2.20	0.16	0.658
C20:5 *n*-3	0.059	0.003	0.060	0.005	0.061	0.004	0.949
C22:5 *n*-3	0.190	0.015	0.164	0.008	0.164	0.013	0.254
C22:6 *n*-3	0.036 ^a^	0.005	0.009 ^b^	0.004	0.003 ^b^	0.002	<0.0001
Σ PUFA	15.5 ^b^	0.5	19.2 ^a^	0.4	20.2 ^a^	0.8	<0.0001
Σ UFA	63.7 ^b^	0.4	65.1 ^a^	0.2	64.1 ^a b^	0.5	0.021
Σ UFA/SFA	1.76 ^b^	0.03	1.87 ^a^	0.02	1.81 ^a b^	0.03	0.023
Σ PUFA/SFA	0.43 ^b^	0.02	0.55 ^a^	0.01	0.57 ^a^	0.03	<0.0001
Σ *n*-3	1.25	0.02	1.32	0.02	1.31	0.03	0.103
Σ *n*-6	14.1 ^b^	0.5	17.8 ^a^	0.4	18.7 ^a^	0.8	<0.0001
Σ *n*-6/*n*-3	11.3 ^b^	0.3	13.5 ^a^	0.3	14.2 ^a^	0.3	<0.0001
Σ *n*-9	41.9 ^a^	0.5	40.6 ^a^	0.3	38.8 ^b^	0.7	0.002
SAT index ^†^	0.56 ^a^	0.01	0.53 ^b^	0.01	0.54 ^a b^	0.01	0.020
ATH index ^‡^	0.45 ^a^	0.01	0.41 ^b^	0.01	0.42 ^b^	0.01	<0.001
Desaturation indexes							
C16:1/C16	0.106 ^a^	0.002	0.090 ^b^	0.004	0.096 ^b^	0.002	0.003
C18:1/C18	3.92 ^a^	0.09	3.75 ^b^	0.09	3.56 ^b^	0.14	0.083
Iodine value ^§^	66.1 ^b^	1.9	70.6 ^a^	0.4	69.5 ^a^	0.5	<0.0001
Peroxidizability index ^ϕ^	22.2 ^b^	0.7	25.9 ^a^	0.6	25.0 ^a^	0.8	0.002

^#^ C and I pigs were fed commercial diets until slaughter, while IExp pigs consumed commercial diets until ~130 kg and then the experimental diet until slaughter; ^a,b^ Values with different superscript letters in the same row are significantly different (*p* < 0.05); ^†^ Saturation index = (C14 + C16 + C18)/(ΣMUFA + ΣPUFA); ^‡^ Atherogenic index = [C12 + (4 × C14) + C16]/(ΣMUFA + Σ*n*-6 + Σ*n*-3); ^§^ Iodine value = 85.703 + (C14 × 2.74) − (C16 × 1.085) − (C:18 × 0.71) + (C18:2 *n*-6 × 0.986); ^ϕ^ Peroxidizability index = (% dienoic) + (% trienoic × 2) + (% tetraenoic × 3) + (% pentaenoic × 4) + (% hexaenoic × 5).

**Table 4:** Main fatty acids profile of dorsal subcutaneous fat from castrated (C), intact (I) and intact experimental (IExp) Alentejano pigs slaughtered at ~160 kg LW (*n* = 10 per group) ^#^.

	C		I		IExp		*p*-Value
	Mean	SE	Mean	SE	Mean	SE	
	g/100 g of total fatty acids identified						
C14	1.27 ^a^	0.03	1.13 ^b^	0.03	1.23 ^a^	0.04	0.006
C16	24.5 ^a^	0.2	23.2 ^b^	0.3	23.3 ^b^	0.2	<0.001
C18	13.6 ^a^	0.3	12.0 ^b^	0.2	11.6 ^b^	0.6	0.004
C20	0.22	0.01	0.20	0.01	0.19	0.01	0.067
Σ SFA	40.2 ^a^	0.3	36.9 ^b^	0.4	36.6 ^b^	0.6	<0.0001
C16:1 *n*-7	1.34	0.07	1.37	0.05	1.51	0.14	0.382
C16:1 *n*-9	0.28 ^b^	0.01	0.46 ^a^	0.03	0.41 ^a^	0.03	<0.0001
C18:1 *n*-7	2.06	0.05	2.14	0.03	2.20	0.12	0.478
C18:1 *n*-9	47.1 ^b^	0.2	48.4 ^a^	0.3	48.3 ^a^	0.3	0.002
Σ MUFA	50.9 ^b^	0.3	52.6 ^a^	0.3	52.7 ^a^	0.6	0.008
C18:2 *n*-6	6.4 ^b^	0.1	7.9 ^a^	0.2	8.0 ^a^	0.2	<0.0001
C18:3 *n*-3	1.86	0.06	1.83	0.07	1.80	0.06	0.751
C20:2 *n*-6	0.41 ^b^	0.02	0.47 ^a^	0.01	0.46 ^a^	0.02	0.037
C20:5 *n*-3	0.007 ^a^	0.001	0.004 ^b^	0.001	0.003 ^b^	0.001	<0.0001
C22:5 *n*-3	0.034 ^a^	0.001	0.016 ^c^	0.001	0.019 ^b^	0.001	<0.0001
C22:6 *n*-3	0.010	0.001	0.008	0.001	0.008	0.001	0.140
Σ PUFA	9.1 ^b^	0.1	10.3 ^a^	0.2	10.7 ^a^	0.2	<0.0001
Σ UFA	59.9 ^b^	0.3	63.1 ^a^	0.4	63.4 ^a^	0.7	<0.0001
Σ UFA/SFA	1.50 ^b^	0.02	1.71 ^a^	0.03	1.73 ^a^	0.05	<0.0001
Σ PUFA/SFA	0.23 ^b^	0.01	0.28 ^a^	0.01	0.29 ^a^	0.01	<0.0001
Σ *n*-3	2.02	0.06	1.98	0.07	1.95	0.07	0.768
Σ *n*-6	6.9 ^b^	0.1	8.4 ^a^	0.2	8.6 ^a^	0.2	<0.0001
Σ *n*-6/*n*-3	3.50 ^b^	0.17	4.20 ^a^	0.25	4.60 ^a^	0.22	0.004
Σ *n*-9	47.4 ^b^	0.2	48.9 ^a^	0.3	48.7 ^a^	0.3	<0.001
SAT index ^†^	0.66 ^a^	0.01	0.58 ^b^	0.01	0.57 ^b^	0.02	<0.0001
ATH index ^‡^	0.50 ^a^	0.01	0.44 ^b^	0.01	0.45 ^b^	0.01	<0.0001
Desaturation indexes							
C16:1/C16	0.054	0.003	0.059	0.002	0.070	0.008	0.056
C18:1/C18	3.47 ^b^	0.09	4.04 ^a^	0.08	4.40 ^a^	0.30	0.001
Iodine value ^§^	59.2 ^b^	1.9	62.8 ^a^	0.4	63.5 ^a^	0.5	<0.0001
Peroxidizability index ^ϕ^	11.4 ^b^	0.2	12.7 ^a^	0.2	12.6 ^a^	0.2	<0.0001

^#^ C and I pigs were fed commercial diets until slaughter, while IExp pigs consumed commercial diets until ~130 kg and then the experimental diet until slaughter; ^a,b,c^ Values with different superscript letters in the same row are significantly different (*p* < 0.05); ^†^ Saturation index = (C14 + C16 + C18)/(ΣMUFA + ΣPUFA); ^‡^ Atherogenic index = [C12 + (4 × C14) + C16]/(ΣMUFA + Σ*n*-6 + Σ*n*-3); ^§^ Iodine value = 85.703 + (C14 × 2.74) − (C16 × 1.085) − (C:18 × 0.71) + (C18:2 *n*-6 × 0.986); ^ϕ^ Peroxidizability index = (% dienoic) + (% trienoic × 2) + (% tetraenoic × 3) + (% pentaenoic × 4) + (% hexaenoic × 5).

**Table 5:** Total phenols, Trolox equivalent antioxidant capacity (TEAC), and ferric reducing antioxidant power (FRAP) of LL from castrated (C), intact (I) and intact experimental (IExp) Alentejano pigs slaughtered at ~160 kg LW (*n* = 10 per group) ^#^.

	C		I		IExp		*p*-Value
	Mean	SE	Mean	SE	Mean	SE	
Total phenols (mg tannic acid equivalents/g)	0.56 ^a^	0.01	0.47 ^b^	0.02	0.45 ^b^	0.01	<0.0001
TEAC (µmol Trolox equivalents/g)	7.56 ^a^	0.38	6.12 ^b^	0.34	6.30 ^b^	0.36	0.017
FRAP (μmol Fe^2+^ equivalents/g)	3.63	0.28	3.24	0.38	3.63	0.24	0.579

^#^ C and I pigs were fed commercial diets until slaughter, while IExp pigs consumed commercial diets until ~130 kg and then the experimental diet until slaughter; ^a,b^ Values with different superscript letters in the same row are significantly different (*p* < 0.05).

## Data Availability

Data will not be shared, due to privacy restrictions.

## Supplementary Materials

The following supporting information can be downloaded at: https://www.mdpi.com/article/10.3390/ani13193099/s1, Table S1: Chemical composition of the commercial and experimental diets consumed by Alentejano pigs slaughtered at ~160 kg LW.
