# Supplementary material for: Influence of Sex on Meat and Fat Quality from Heavy Alentejano Pigs Finished Outdoors on Commercial and High Fiber Diets
Source: Animals (Basel). 2023 Oct 4;13(19):3099. doi: 10.3390/ani13193099 (PMC10571643; doi:10.3390/ani13193099)
Supplement: Supplementary file 1 [file animals-13-03099-s001.zip › animals-2584981-supplementary -new-10.2/Animals_ECO-PIG Alentejanos_PM_DSF_Table S1.pdf]

Table S1. Chemical composition of the commercial and experimental diets consumed by Alentejano pigs slaughtered at ~160 kg LW

	**Growing diet A**[1]	**Growing diet B**[2]	**Fattening diet**[3]	**Experimental diet**[4]
Dry matter (DM) (g/100 g)	90.7	91.8	90.8	90.5
			(g/100 g DM)	
Total ashes	7.6	7.1	8.1	8.2
Crude protein (N x 6.25)	16.8	16.0	13.8	13.8
Lysine[5]	0.73	0.59	0.47	0.48
Methionine[5]	0.24	0.24	0.20	0.18
Cysteine + cystine[5]	0.29	0.29	0.26	0.22
Tryptophan[6]	0.20	0.20	0.16	0.14
Total fiber[7]	17.4	23.6	26.9	40.2
Insoluble fiber[7]	6.1	13.1	15.1	28.0
Neutral detergent fiber (NDF)	24.1	25.3	22.6	29.9
Acid detergent fiber (ADF)	7.3	11.1	9.4	13.2
Acid detergent lignin (ADL)	0.74	1.59	1.64	0.90
Hemicellulose[8]	16.8	14.2	13.2	16.7
Cellulose[9]	5.5	9.1	6.5	10.4
Total sugars[10]	3.5	3.1	3.0	1.88
Total starch[11]	47.0	51.8	45.5	40.1
Total lipids	3.0	4.0	6.8	6.2
Palmitic acid (C16:0)	0.44	0.45	0.66	0.66
Oleic acid (C18:1 *n*-9)	1.88	3.03	4.42	5.41
Linoleic acid (C18:2 *n*-6)	1.33	1.01	1.66	1.55
Saturated fatty acids	0.44	0.56	0.88	0.88
Monounsaturated fatty acids	1.88	3.03	4.42	5.41
Polyunsaturated fatty acids	1.33	1.01	1.77	1.66
Σ *n*-3	0.09	0.10	0.14	0.15
Σ *n*-6	1.20	0.94	1.46	1.39
			(MJ/kg)	
Digestible energy[12]	13.6	13.2	13.2	13.1

Notes: [1]Fed from 40 to 60 kg LW to all experimental groups; [2]Fed from 60 to 120 kg to all experimental groups; [3]Fed from 120 to 160 kg to C and I, and from 120 to 130 kg to IExp group; [4]Fed from 130 to 160 kg to IExp group; [5][20]; [6][21]; [7][22] method 991.43; [8]Hemicellulose = NDF-ADF; [9][23]; [10][22] method 982.14; [11][24]; [12][25].

References:

20. ISO-13903. Animal feeding stuffs - Determination of amino acids content. **2005**, *ISO 13903*, 17.
21. European Commission. Commission Regulation (EC) No 152/2009 of 27 January 2009 laying down the methods of sampling and analysis for the official control of feed. *Official Journal of the European Union* **2009**, *L 54*, 1-169.
22. AOAC. *Official Methods of Analysis of AOAC International*, 18th ed.; AOAC: Gaithersburg, MD, 2006.

23. ISO-6865. Animal feeding stuffs - Determination of crude fibre content - Method with intermediate filtration. **2000**, *ISO 6865*, 10.
24. ISO-6493. Animal feeding stuffs - Determination of starch content - Polarimetric method. **2000**, *ISO 6493*, 10.
25. Noblet, J.; Fortune, H.; Dupire, C.; Dubois, S. Digestible, metabolizable and net energy values of 13 feedstuffs for growing pigs: effect of energy system. *Animal Feed Science and Technology* **1993**, *42*, 131-149.
